# Resting-state functional connectivity patterns associated with childhood maltreatment in a large bicentric cohort of adults with and without major depression

**DOI:** 10.1017/S0033291722001623

**Published:** 2023-07

**Authors:** Janik Goltermann, Nils Ralf Winter, Susanne Meinert, Lisa Sindermann, Hannah Lemke, Elisabeth J. Leehr, Dominik Grotegerd, Alexandra Winter, Katharina Thiel, Lena Waltemate, Fabian Breuer, Jonathan Repple, Marius Gruber, Maike Richter, Vanessa Teckentrup, Nils B. Kroemer, Katharina Brosch, Tina Meller, Julia-Katharina Pfarr, Kai Gustav Ringwald, Frederike Stein, Walter Heindel, Andreas Jansen, Tilo Kircher, Igor Nenadić, Udo Dannlowski, Nils Opel, Tim Hahn

**Affiliations:** 1 University of Münster, Institute for Translational Psychiatry, Münster, Germany; 2 University of Münster, Institute for Translational Neuroscience, Münster, Germany; 3 Department of Psychiatry and Psychotherapy, University of Tübingen, Tübingen, Germany; 4 Department of Psychiatry & Psychotherapy, University of Bonn, Bonn, Germany; 5 Department of Psychiatry, University of Marburg, Marburg, Germany; 6 University of Münster, Department of Clinical Radiology, Münster, Germany; 7 University of Münster, Interdisciplinary Centre for Clinical Research (IZKF), Münster, Germany; 8 Department of Psychiatry and Psychotherapy, Jena University Hospital, Jena, Germany

**Keywords:** adverse childhood experiences, amygdala, childhood maltreatment, depression, emotion regulation, resting-state functional connectivity

## Abstract

**Background:**

Childhood maltreatment (CM) represents a potent risk factor for major depressive disorder (MDD), including poorer treatment response. Altered resting-state connectivity in the fronto-limbic system has been reported in maltreated individuals. However, previous results in smaller samples differ largely regarding localization and direction of effects.

**Methods:**

We included healthy and depressed samples [*n* = 624 participants with MDD; *n* = 701 healthy control (HC) participants] that underwent resting-state functional MRI measurements and provided retrospective self-reports of maltreatment using the Childhood Trauma Questionnaire. A-priori defined regions of interest [ROI; amygdala, hippocampus, anterior cingulate cortex (ACC)] were used to calculate seed-to-voxel connectivities.

**Results:**

No significant associations between maltreatment and resting-state connectivity of any ROI were found across MDD and HC participants and no interaction effect with diagnosis became significant. Investigating MDD patients only yielded maltreatment-associated increased connectivity between the amygdala and dorsolateral frontal areas [*p*_FDR_ < 0.001; *η*^2^_partial_ = 0.050; 95%-CI (0.023–0.085)]. This effect was robust across various sensitivity analyses and was associated with concurrent and previous symptom severity. Particularly strong amygdala-frontal associations with maltreatment were observed in acutely depressed individuals [*n* = 264; *p*_FDR_ < 0.001; *η*^2^_partial_ = 0.091; 95%-CI (0.038–0.166)). Weaker evidence – not surviving correction for multiple ROI analyses – was found for altered supracallosal ACC connectivity in HC individuals associated with maltreatment.

**Conclusions:**

The majority of previous resting-state connectivity correlates of CM could not be replicated in this large-scale study. The strongest evidence was found for clinically relevant maltreatment associations with altered adult amygdala-dorsolateral frontal connectivity in depression. Future studies should explore the relevance of this pathway for a maltreated subgroup of MDD patients.

## Introduction

Childhood maltreatment (CM) has been identified as a major risk factor for the development of various mental health disorders, particularly major depressive disorder (MDD) (Nemeroff, 2016). Depressive patients with maltreatment experiences show a higher likelihood of a chronic disease trajectory as well as poorer treatment outcomes, pointing to a distinct psychopathological phenotype (Nanni, Uher, & Danese, 2012; Opel et al., 2019; Teicher & Samson, 2013). Understanding the neurobiological consequences of CM as risk factor is crucial to develop early and effective therapeutic interventions (Teicher, Samson, Anderson, & Ohashi, 2016). Against this backdrop, previous research indicates that extreme or enduring stress during childhood leads to long-lasting neurobiological changes in the stress-processing system (Nemeroff, 2016). These changes might be marked by an altered brain structure and function, especially affecting prefrontal and limbic areas (Dannlowski et al., 2013, 2012; Frodl et al., 2017; Heany et al., 2018; Kessler et al., 2020; Lim, Radua, & Rubia, 2014; Opel et al., 2019, 2016; Popovic et al., 2020; Teicher et al., 2016; Tozzi et al., 2020). Paralleling effects of CM, structural and functional neural correlates of depression have been localized in similar regions such as the anterior cingulate cortex (ACC), the hippocampus or the amygdala (Gray, Müller, Eickhoff, & Fox, 2020). This observed overlap in neural correlates of depression and CM has nourished the interpretation that brain alterations could play an important role in the link between CM and depression (Meinert et al., 2019; Opel et al., 2014; Teicher & Samson, 2013).

In addition to alterations in gray matter structure and function, research on brain connectivity and its putative role for psychopathology has received increased attention in recent years (Kovner, Oler, & Kalin, 2019). Particularly, the connectivity between frontal and limbic regions has been implied in emotion regulation, thus taking a central role in neurobiological models of affective disorders (Dixon, Thiruchselvam, Todd, & Christoff, 2017). Studies investigating resting-state functional connectivity (i.e. synchronization of the spontaneous fluctuations of activity between brain regions during rest) support this notion, linking patterns of functional connectivity to CM (Wang et al., 2014), as well as depression (Iwabuchi et al., 2015; Mulders, van Eijndhoven, Schene, Beckmann, & Tendolkar, 2015). More specifically, MDD patients have shown lower subgenual ACC connectivity and higher amygdala connectivity with other subcortical areas (Oathes, Patenaude, Schatzberg, & Etkin, 2015). In addition, functional connectivity of different frontal and limbic regions has been shown to predict antidepressant treatment outcomes (Chin Fatt et al., 2020; van Waarde et al., 2015).

Paralleling resting-state findings in depression research, altered fronto-limbic functional connectivity has also been observed in individuals with a history of CM, both in clinical and non-clinical samples. In a community sample of *n* = 64 late-adolescents a higher burden of CM experiences was associated with lower connectivity of amygdala and hippocampus seed regions, specifically with the sgACC (Herringa et al., 2013). In contrast, a specific investigation of CM associations with the sgACC in a sample of healthy adolescents (*n* = 68) yielded a weaker positive connectivity with the dorsolateral prefrontal cortex (dlPFC), supramarginal gyrus and cuneus, but failed to replicate an effect involving the amygdala or hippocampus (Hoffmann et al., 2018). Others have shown that CM is negatively associated with the connectivity of amygdala and hippocampus seed regions with the prefrontal cortex (PFC), including portions of the ACC (*n* = 27) (Birn, Patriat, Phillips, Germain, & Herringa, 2014). Similarly, van der Werff et al., reported on reduced positive amygdala-orbitofrontal cortex connectivity associated with emotional maltreatment in a mixed sample with and without psychiatric disorders (*n* = 88) (van der Werff et al., 2013). In contrast, Kaiser et al., found increased negative connectivity between amygdala and dlPFC but no connectivity alterations of ACC or hippocampus associated with CM in a sample of n = 70 adult individuals with affective and anxiety disorders (Kaiser et al., 2018). To sum up, altered fronto-limbic functional connectivity in individuals with CM experiences could represent a neurobiological link to psychiatric symptoms (Teicher et al., 2016).

However, despite some degree of replicability of altered connectivity in frontal and limbic areas (most prominently amygdala, hippocampus, and PFC regions including the ACC), findings differ regarding the specific regions involved. Even within similar regions, inconsistencies with respect to the direction of CM effects are evident in the current literature. While the findings cited above indicate a negative association between CM and fronto-limbic connectivity (resulting either in a decreased correlation or increased anticorrelation between brain regions), others have reported on opposite effects (Jedd et al., 2015; Thomason et al., 2015). Furthermore, it should be noted that some studies have failed to replicate fronto-limbic connectivity alterations associated with CM altogether and tentatively point to effects located elsewhere – e.g. connectivity alterations of the cerebellum, precuneus, posterior cingulate cortex and fusiform gyrus (Boccadoro et al., 2019; Philip et al., 2013b).

Heterogeneity within the current literature may result from several methodological aspects. First, study samples differed massively regarding the degree of maltreatment experiences, presence of clinical diagnoses, as well as in operationalizations of CM. While most of the above studies conduct cutoff-based group comparisons of CM *v.* non-CM samples (Boccadoro et al., 2019; Hoffmann et al., 2018; Jedd et al., 2015; Philip et al., 2013a; Thomason et al., 2015; van der Werff et al., 2013), others have employed a continuous rather than categorical operationalization, thus expressing CM severity (Birn et al., 2014; Herringa et al., 2013; Kaiser et al., 2018). It is yet unclear how such differences in operationalizations of CM could impact the findings, although continuous conceptualizations may implicate higher psychometric qualities (Goltermann et al., 2021).

Second, although some studies include psychiatric as well as healthy participants, a systematic investigation of the role of MDD to CM-related functional connectivity effects is yet missing. Likewise, the current clinical state of individuals with a psychiatric disorder could pose another source of heterogeneity, as evidence suggests that resting-state connectivity differs largely across acute and remitted bipolar (Wang, Feng, Mitchell, Wang, & Si, 2020a, 2020b) and unipolar depressive patients (Wang et al., 2020a). Third, previous studies are based on small samples (with the vast majority reporting on *N* < 70) and are likely to be underpowered considering the common small effect sizes in the neuroimaging domain (Elliott et al., 2020). Despite some reports of large effect sizes in single studies (Birn et al., 2014), small sample sizes suggest that those could be overestimated (Kühberger, Fritz, & Scherndl, 2014). Therefore, it is crucial to secure previous findings in a sufficiently large sample to reevaluate our knowledge on the effects of CM on functional connectivity within the adult brain.

In this study, we therefore aim to shed light on the current heterogeneity of findings and investigate the association between CM and resting-state functional connectivity in an unprecedented large adult cohort of individuals both with and without MDD. Based on the literature presented above, we test the hypotheses of CM-associated altered resting-state connectivity of the (1) amygdala, (2) hippocampus, and (3) ACC as seed regions of interest (ROIs), investigating their whole-brain voxel-wise connectivity. We conducted joint as well as subgroup analyses for healthy control (HC) and MDD samples to compare effects with previously published findings obtained from clinical and non-clinical samples. Further, we explore the impact of differential CM operationalizations as well as current remission status on resting-state effects.

## Materials and methods

### Participants and design

A total of *n* = 624 MDD and *n* = 701 HC were included in the current analysis (age 18–65, mean age 36.17 years, s.d. 13.06; 65.40% female), as part of the Marburg Münster Affective Disorders Cohort study (MACS) that has been extensively described elsewhere (Kircher et al., 2018; Vogelbacher et al., 2018). Clinical and demographic sample characteristics are displayed in online Supplementary Table S1. For exclusion criteria, see the online Supplementary Material. Participants were recruited at the Departments of Psychiatry at the university hospitals in Münster and Marburg, Germany. They received financial compensation and gave written and informed consent. The MACS was approved by the ethics committees of the Medical Faculties, University of Marburg (AZ: 07/14) and University of Münster (AZ: 2014-422-b-S).

### Clinical and maltreatment measures

The German 28-item version of the Childhood Trauma Questionnaire (CTQ) (Wingenfeld et al., 2010) was administered to assess CM experienced before age 18. The CTQ is a retrospective self-report questionnaire assessing different types of CM. The questionnaire has been widely used across a variety of countries for the assessment of CM experiences in clinical and non-clinical samples and has been extensively validated in different languages (Bernstein et al., 1994, 2003; Karos, Niederstrasser, Abidi, Bernstein, & Bader, 2014; Spinhoven et al., 2014; Viola et al., 2016). The sum score can be used to represent an overall maltreatment severity. Categorical cutoffs defined by Walker and colleagues were used to categorize participants into maltreated and non-maltreated for additional analyses (see online Supplementary Material) (Walker et al., 1999). Information on the categorical prevalence of CM subtypes across HC and MDD participants can be found in online Supplementary Table S3.

Psychiatric diagnoses or the lack thereof was confirmed by trained personnel using the Structural Clinical Interview for DSM-IV-TR (SCID-I) (Wittchen, Wunderlich, Gruschwitz, & Zaudig, 1997). According to the SCID-I, participants were classified as HC (not fulfilling lifetime criteria for any psychiatric diagnosis) and MDD (fulfilling lifetime MDD criteria). Further, a subset of lifetime MDD participants that additionally fulfilled the criteria of a current depression were denoted as acute MDD (*n* = 265). A detailed description of psychiatric comorbidities based on the SCID-I can be found in online Supplementary Table S2. For clinical characterization current symptomatology was assessed using the self-report Beck depression inventory (BDI) (Beck, Ward, Mendelson, Mock, & Erbaugh, 1961), the global level of functioning was assessed by trained raters based on the DSM-IV (Saß, Wittchen, Zaudig, & Houben, 2003), and the previous course of disease was assessed via self-reported number of psychiatric hospitalizations.

### Resting-state fMRI acquisition and preprocessing

MRI data were acquired using a 3T whole body MRI scanner (Marburg: Tim Trio, Siemens, Erlangen, Germany; Münster: Prisma, Siemens, Erlangen, Germany). Due to the two scanner sites and a change of the scanner body-coil in Marburg during recruitment, two dummy variables for three different scanner specifications were used as covariates in all analyses. Resting-state functional images were acquired over a duration of eight minutes (eyes closed). Preprocessing was done with the CONN (v18b) MATLAB toolbox using the default volume-based MNI preprocessing pipeline (https://web.conn-toolbox.org/) (Whitfield-Gabrieli & Nieto-Castanon, 2012) and SPM12 (Welcome Department of Cognitive Neurology, London, UK; https://www.fil.ion.ucl.ac.uk/spm/). Additional information on scanner sequence, preprocessing parameters and quality check procedures are presented in the online Supplementary Materials.

### Selection of regions of interest

Based on the previous literature we identified the amygdala, hippocampus, and ACC as most prominent regions with repeatedly reported CM effects on resting-state connectivity. As previous findings suggest a functional distinction of different parts of the ACC (Rolls, Huang, Lin, Feng, & Joliot, 2020), we applied distinct region of interest (ROI) masks for the subgenual, pregenual and the supracallosal ACC (sgACC, pgACC, scACC). We decided against the definition of further PFC ROIs due to substantial uncertainty regarding the exact localization of PFC effects based on previous findings outlined above (involving medial, dorsolateral and orbitofrontal regions of the PFC, overall covering a large area). Thus, we limited our ROIs to amygdala, hippocampus and ACC regions in a seed-to-voxel approach, which however is also sensitive to identify potential connectivity effects between selected ROIs and PFC regions.

The outlined ROIs were taken from the Automated Anatomical Labelling Atlas version 3 (AAL3) (Rolls et al., 2020) to create ROI masks for seed-based analyses. In order to accommodate for occasional lateralized resting-state findings (Hoffmann et al., 2018; Philip et al., 2014; van der Werff et al., 2013), we applied each ROI for the left and right hemisphere separately, resulting in a total of 10 ROIs (ROIs are depicted in online Supplementary Fig. S1). For each seed ROI the fMRI signal was averaged across voxels resulting in one signal time series per ROI. Pearson correlations between each seed ROI time series and each whole-brain voxel time series were conducted as a measure of functional connectivity (seed-to-voxel analysis).

### Statistical analyses

A general linear model was applied to investigate the main effects of CTQ sum, controlling for age, sex, and two scanner condition dummies. In a first step, the pooled sample (HC and MDD) was investigated for the main effects of CM, as well as an interaction of CM with MDD diagnosis. Within the pooled sample, the diagnosis was added as an additional control variable. Subsequently, HC and MDD samples were investigated separately for two reasons: Firstly, this was done due to substantial differences in CTQ sum distributions across HC and MDD subgroups with regard to mean, variance and skewness (see online Supplementary Fig. S2), thus raising concerns about the validity of joint analysis of both heterogeneous subgroups in one statistical model. We further ran separate analyses for a subset of the MDD sample currently fulfilling the criteria for an acute depressive episode to account for the findings of differing resting-state connectivity during the presence of concurrent affective symptoms outlined above (Wang et al., 2020a, 2020b). The second reason for subgroup analysis is that it enables us to control for medication effects within the MDD group that may influence limbic connectivity (McCabe & Mishor, 2011). Follow-up analyses additionally controlling for psychiatric medication, educational attainment, and motion parameters, as well as exploring the role of sex and maltreatment subtypes were conducted for effects significant at a Bonferroni-corrected level at *p* < 0.05. Psychiatric medication load was operationalized by calculating a medication index incorporating the number and dosage of concurrent medication (described in more detail within the supplements), educational attainment was operationalized as the reported years of education, and the individual mean motion was used as a motion covariate. The CTQ subscale scores were separately used as predictors instead of CTQ sum to probe CM subtype effects.

Further, exploratory analyses with different CM operationalizations were conducted and are reported within the online Supplementary Results (investigating categorical definitions and extreme forms of CM).

For connectivity analyses a combination of a False Discovery Rate (FDR)-corrected cluster threshold of *p* < 0.05 and an uncorrected voxel threshold of *p* < 0.001 were used as adjunctive significance criteria. Effects were tested using two-sided contrasts due to inconsistent findings regarding the direction of CM associations with resting-state connectivity. FDR-corrected cluster-size *p* values and *partial η^2^* as cluster-level effect size measure are reported with all significant results. Due to non-normality of the CTQ data we calculated non-parametric bias corrected accelerated bootstrapped confidence intervals for all effect sizes (*N* = 1000 bootstrap samples). This method accounts for the skewness of the bootstrapped data and has been shown to produce robust and valid confidence intervals under non-normality circumstances (Davison & Hinkley, 1997; Kelley, 2005). In addition to within-analysis cluster-size FDR-correction, we further computed and report Bonferroni-corrected *p* values controlling for the false-positive rate across the number of ROIs (implying correction for ten tests).

Achieved statistical sensitivity (i.e. the minimum required effect size to detect a main effect of CTQ) was computed for the available samples using g*Power software (Faul, Erdfelder, Lang, & Buchner, 2007). For a statistical power of 95% and *α* = 0.001, required effect sizes were *η*^2^_partial_ = 0.018 for the whole sample, *η*^2^_partial_ = 0.034 for the HC sample, *η*^2^_partial_ = 0.038 for the lifetime MDD sample, and *η*^2^_partial_ = 0.086 for the acute MDD sample (details within the supplements).

## Results

### Resting-state functional connectivity correlates of childhood maltreatment

#### Pooled clinical and non-clinical sample

Testing a CTQ sum effect in the whole sample (pooling MDD and HC participants together) yielded no significant suprathreshold clusters for any of the ten seed ROIs, neither with nor without multiple-ROI correction. Testing an interaction effect between CTQ sum and diagnosis yielded no significant effects for any of the ten seed ROIs on a Bonferroni-corrected level. Applying a more lenient threshold without correcting for multiple ROI analyses yielded an interaction effect on the connectivity between the left sgACC and a cluster in the right superior temporal region (*p*_FDR_ = 0.018), but no interaction effect for any of the other nine ROIs.

#### Sample with lifetime MDD diagnosis

Within lifetime MDD participants, higher CTQ sum scores were associated with higher positive connectivity of the right amygdala ROI with a single cluster in the dlPFC (*p_FDR_* < 0.001, located mainly within the right superior and middle frontal gyrus, corresponding to Brodmann areas 8, 6 and 9, Table 1). This effect remained significant when applying Bonferroni correction for multiple ROI analyses.

**Table 1.** Significant clusters with main effects of continuous childhood maltreatment operationalization (CTQ sum) in ROI-based seed-to-voxel analyses

Sample	*N*	ROI	Main regions of significant cluster	*k*	Peak-voxel coordinates	Direction of effect	*p*-FDR	*p*-FDR_Bonferroni_	partial *η*^2^ (BCa 95%-CI)
HC	701	scACC R	Middle temporal L	107	−54 −39 + 3	positive	0.005	0.052	0.032 (0.013–0.059)
		scACC R	Medial superior frontal L + R	133	−9 + 42 + 36	positive	0.008	0.084	0.030 (0.011–0.060)
		scACC R	Middle temporal R, Superior temporal R	96	+ 60 + 9 −18	positive	0.009	0.094	0.032 (0.010–0.061)
		Hippocampus L	Cerebellum R	67	+ 30 −45 −42	negative	0.049	0.494	0.038 ( 0.011–0.081)
MDD lifetime	624	**Amygdala R**	**Superior frontal R, Middle frontal R**	**197**	**+ 21 + 45 + 48**	**positive**	**<0.001**	**0.006**	**0.050 (0.023–0.085)**
		Amygdala R	Fusiform R	70	+ 24 −9 −21	positive	0.036	0.364	0.035 (0.015–0.066)
		Amygdala R	Middle cingulate L + R	87	0 −36 + 48	positive	0.024	0.240	0.032 (0.012–0.063)
MDD acute	264	**Amygdala R**	**Superior frontal R, Middle frontal R**	**168**	**+ 51 + 21 + 45**	**positive**	**<0.001**	**0.007**	**0.091 (0.038–0.166)**

HC, healthy control; MDD, major depressive disorder; CTQ, childhood trauma questionnaire; CM, childhood maltreatment; sc, supracallosal; ACC, anterior cingulate cortex; L, left; R, right; CI, confidence interval.

*Note.* FDR-corrected cluster-size *p* values are presented with and without additional Bonferroni correction. Results in bold font are still significant after Bonferroni correction across multiple region of interest analyses. The direction of effects indicates if the association between CTQ sum and seed-to-voxel connectivity is positive (higher positive or less negative connectivity with higher CTQ sum scores) or negative (higher negative or less positive connectivity with higher CTQ sum scores). Bootstrapped (*N* = 1000 samples) bias-corrected and accelerated (BCa) 95% confidence intervals are presented for all effect sizes.

**Fig. 1.**
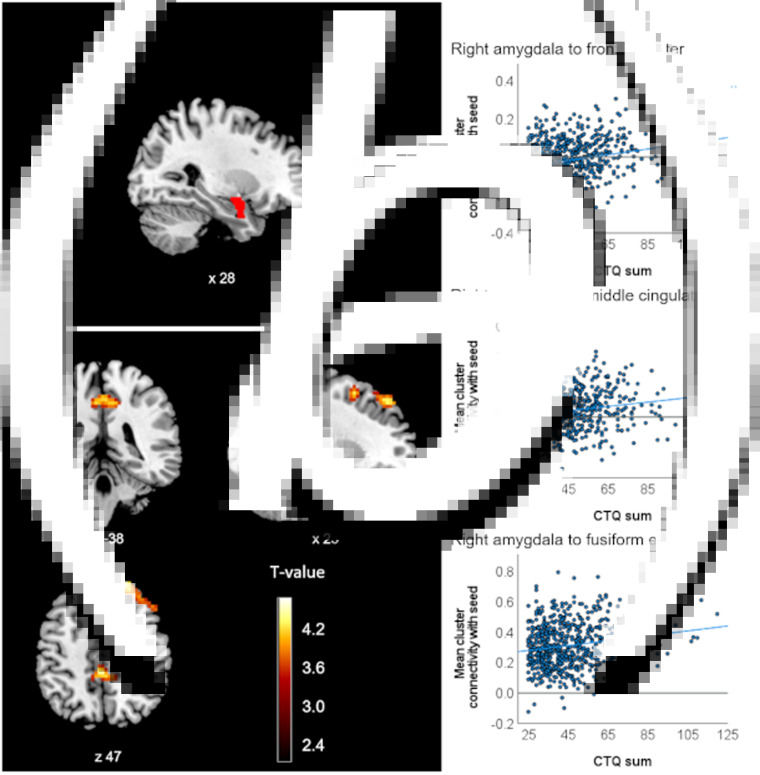
Clusters with altered connectivity to the right amygdala associated with CTQ sum in lifetime MDD participants. (a) Right amygdala seed region of interest (ROI) presented in red. (b) Significant clusters (before Bonferroni correction) with altered connectivity associated with maltreatment. Slice position is displayed using MNI-coordinates. (c) Scatter plots with individual CTQ sum scores and connectivity values between seed ROI and significant clusters.

Connectivity of the right amygdala with two additional clusters in the right fusiform gyrus (*p_FDR_* *=* 0.036) and bilateral middle cingulate cortex (*p_FDR_* = 0.036) were also positively associated with CTQ sum, but did not survive Bonferroni correction for multiple ROI analyses (Table 1). Effect size estimates of the positive relationship between CTQ sum and the observed amygdala connectivities ranged from *η*^2^_partial_ = 0.032 to 0.050 (Table 1). CM-associated amygdala connectivities are presented in Fig. 1. No significant associations were observed in this sample between CTQ sum and the connectivity of the left amygdala or any hippocampus or ACC ROI (also not for the left sgACC where the interaction with the diagnosis was found in the pooled sample).

As the effect of CTQ sum on right amygdala connectivity to the dlPFC cluster in the lifetime MDD sample was the only effect among all primary planned analyses that survived Bonferroni-correction for multiple comparisons, we followed up on this main finding with additional robustness analyses. This finding was not qualitatively changed when controlling for additional covariates (medication index, educational attainment, mean motion), applying Threshold-free Cluster Enhancement as an alternate approach to control for false-positives, or when excluding participants with a comorbid eating disorder, substance use disorder, or psychotic disorder (see online Supplementary Results). On a descriptive level, the largest effect sizes for the amygdala-dlPFC connectivity were observed for the emotional abuse and physical neglect subscales, with the lowest effect size obtained for sexual abuse (see supplements, online Supplementary Table S4). CTQ subscales were strongly correlated (supplements, online Supplementary Table S9). In order to investigate the robustness of the finding of altered amygdala connectivity we further applied an alternate more conservative motion thresholding, resulting in a reduced sample of *n* = 569 MDD participants with at least 5 min of non-scrubbed resting-state scans. This alternate analysis yielded three clusters with a positive effect of CTQ sum on right amygdala connectivity: a cluster in the left hippocampal and temporal regions, a cluster left and right middle cingulate cortex, precuneus and paracentral lobule as well as one cluster located in the right (para)hippocampal and fusiform region. However, the cluster in the right dlPFC did not reach significance using this alternate motion thresholding (supplements, online Supplementary Table S8).

In an exploratory analysis in the MDD subsample displaying acute depressive symptomatology (*n* = 264), the positive relationship between the CTQ sum and right amygdala connectivity with the reported cluster in the dlPFC was confirmed (*p_FDR_* < 0.001, *η^2^_partial_* = 0.091). This effect also survived the Bonferroni correction.

#### Sample without a history of psychiatric disorders

**Fig. 2.**
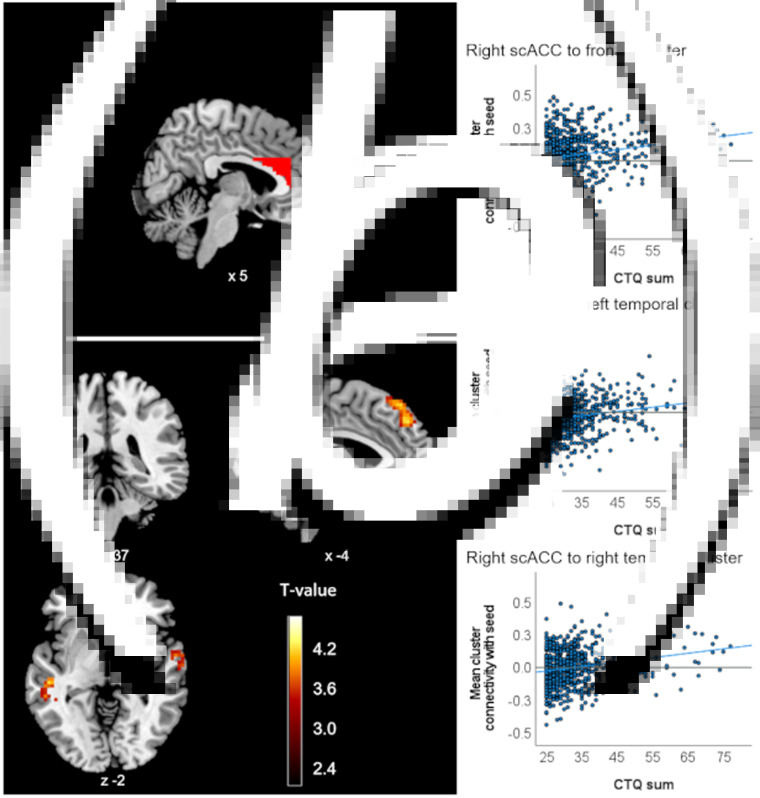
Clusters with altered connectivity to the scACC associated with CTQ sum in healthy controls. (a) Right scACC seed region of interest (ROI) presented in red. (b) Significant clusters (before Bonferroni correction) with altered connectivity associated with maltreatment. Slice position is displayed using MNI-coordinates. (c) Scatter plots with individual CTQ sum scores and connectivity values between seed ROI and significant clusters.

**Fig. 3.**
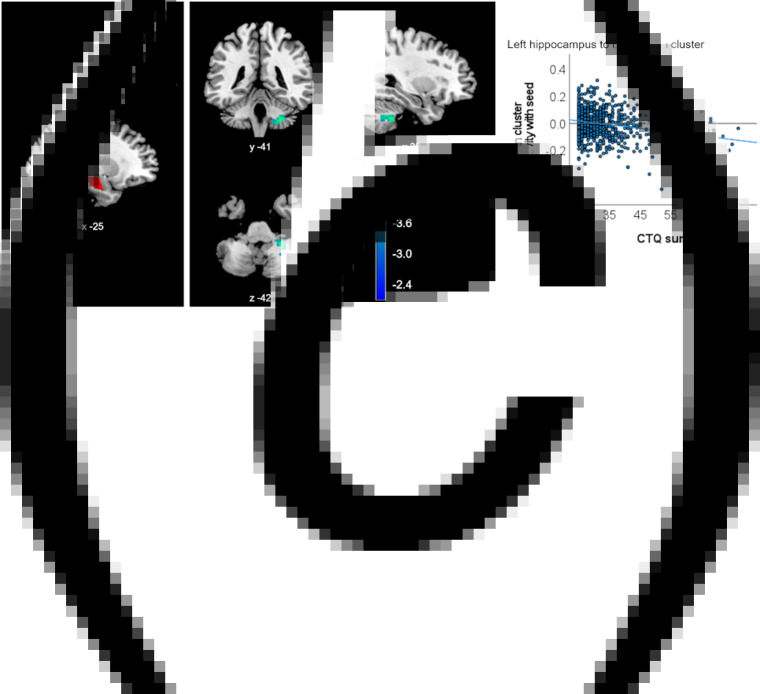
Clusters with altered connectivity to the left hippocampus associated with CTQ sum in healthy controls. (a) Left hippocampus seed region of interest (ROI) presented in red. (b) Significant clusters (before Bonferroni correction) with altered connectivity associated with maltreatment. Slice position is displayed using MNI-coordinates. (c) Scatter plot with individual CTQ sum scores and connectivity values between seed ROI and significant cluster.

In HC, no CM effects on ROI connectivity survived Bonferroni correction. However, applying a more lenient significance threshold without correcting for multiple ROI analyses yielded an association of CTQ sum with higher positive connectivity of the right scACC ROI and three clusters in the bilateral temporal and superior frontal gyrus (left temporal: *p_FDR_* = 0.005, frontal: *p_FDR_* = 0.008, right temporal: *p_FDR_* = 0.009, Table 1, Fig. 2), as well as with higher negative connectivity between the left hippocampus ROI and a cluster in the cerebellum (*p_FDR_* = 0.049, Fig. 3). CTQ sum accounted for about three percent of the variance in reported ROI-cluster connectivities (*η*^2^_partial_ = 0.030 to 0.038, Table 1). CTQ sum showed no significant connectivity associations in this sample with the right hippocampus, either amygdala ROI or any of the ACC ROIs (also not for the left sgACC where the interaction was found).

#### Connectivity analyses using different maltreatment operationalizations

Categorical group comparisons of cutoff-defined CM *v.* non-CM and between groups with extreme CM *v.* non-CM were mostly consistent with CTQ sum analyses, although overall the CTQ sum analyses seemed somewhat more sensitive to detect CM effects. As for the CTQ sum analyses, other operationalizations of CM yielded no main effects when pooling the sample. Occasional main effects were found within subgroup analyses when applying a lenient significance threshold without correction for multiple ROI analyses. With additional Bonferroni correction none of the combinations between ROIs and CM operationalizations yielded significant results. Results are presented in the online Supplementary Table S6.

### Clinical correlates of maltreatment-associated fronto-limbic connectivity in depression

**Fig. 4.**
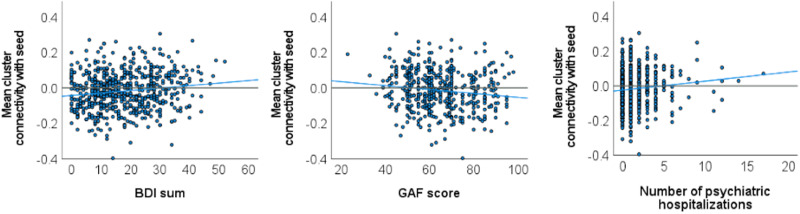
Scatter plots of associations between individual amygdala-frontal connectivity and clinical measures within the MDD sample. Individual amygdala seed connectivities with frontal cluster significantly associated with childhood maltreatment are displayed on the *y*-axis. BDI, Beck depression inventory; GAF, global assessment of functioning.

Exploratory analyses were conducted to investigate the potential clinical relevance of CM-related connectivity effects in the MDD sample. To this end we extracted individual connectivity values of the amygdala-dlPFC cluster significantly associated with CTQ sum in our analysis and correlated them with measures of acute illness severity and previous course of disorder. Higher connectivity between the amygdala and the frontal cluster was significantly associated with higher concurrent depression severity [BDI, rho = 0.141, BCa 95%-CI (0.061–0.219), *p* < 0.001, *n* = 616], lower global levels of functioning [GAF, rho = −0.152, BCa 95%-CI (−0.058 to −0.258), *p* = 0.001, *n* = 497), and a higher number of previous psychiatric hospitalizations (rho = 0.102, BCa 95%-CI (0.023–0.174), *p* = 0.011, *n* = 615] (Fig. 4). Further, higher connectivity between the amygdala and the right fusiform cluster was associated with higher BDI scores [rho = 0.090, BCa 95%-CI (0.003–0.173), *p* = 0.026, *n* = 616]. All other correlations between CM-associated amygdala connectivity to the fusiform cluster, as well as to the middle cingulate gyrus with clinical measures were non-significant (all *p* > 0.079).

## Discussion

In this study, we present findings on fronto-limbic functional connectivity in adults with a history of CM in an unprecedented large sample of healthy and depressive individuals. While the majority of previous resting-state connectivity correlates of maltreatment could not be replicated in this study, subgroup analyses suggest that maltreatment experiences could be associated with higher fronto-limbic connectivity in MDD patients. However, our findings also indicate that these correlates of maltreatment are more subtle than suggested by earlier studies and do not generalize across clinical and non-clinical samples, potentially explaining the heterogeneous and often contradictory reports in the previous literature.

We did not observe a CM effect when pooling healthy and depressed samples despite large statistical power. Testing various maltreatment definitions rule out that obtained null-findings were produced by a specific CM operationalization. Even investigating subgroups with severe levels of CM did not change this pattern of results. Taken together, these findings contradict previously published associations between experiences of CM and resting-state functional connectivity of the investigated regions (Birn et al., 2014; Herringa et al., 2013; Hoffmann et al., 2018; Kaiser et al., 2018; van der Werff et al., 2013). One possible explanation could be that previous small-scale studies have been reporting false-positive results.

When analyzing the MDD sample only, evidence for altered fronto-limbic connectivity emerged. Individuals with a lifetime MDD displayed effects of CM on resting-state functional connectivity of the amygdala to frontal regions but not the hippocampus or any ACC region. More specifically, CM severity was associated with higher positive connectivity of the right amygdala with a cluster located in the right superior and middle frontal gyrus. This effect showed a modest effect size and was the only connectivity effect surviving correction for multiple testing. This frontal cluster covers an area often referred to as the dlPFC, which has been implied to be a key region for emotion regulation processes (Dixon et al., 2017; Phillips, Ladouceur, & Drevets, 2008). Neurobiological models of fronto-limbic emotion regulation postulate that the dlPFC reflects on, appraises and if necessary downregulates emotional responses of the amygdala (Ochsner, Bunge, Gross, & Gabrieli, 2002). Altered amygdala-dlPFC connectivity has been repeatedly suggested to constitute a neural correlate of emotion dysregulation which is a symptom often observed in children (Gruhn & Compas, 2020; Kim & Cicchetti, 2010) and adults (Burns, Jackson, & Harding, 2010) with a history of CM. Emotion dysregulation has further been proposed to be one of the potential pathways between CM and depression (O'Mahen, Karl, Moberly, & Fedock, 2015). Better recruitment of the dlPFC during an emotion regulation task is correlated with higher amygdala downregulation and less severe depressive symptoms two years later in children with maltreatment experiences (Rodman, Jenness, Weissman, Pine, & McLaughlin, 2019). Weak but significant correlations between amygdala-dlPFC connectivity and clinical variables in our study support the interpretation that this CM effect might be clinically meaningful. We provide evidence that the CM-related altered connectivity can be associated with current depression severity, global level of functioning and previous disease severity, as indicated by the frequency of psychiatric hospitalizations. Further, the association between CM and amygdala-dlPFC connectivity was aggravated within MDD participants experiencing an acute depressive episode, which could point to state-dependent relevance of neurobiological emotion regulation abnormalities. The dlPFC is part of a network involved in emotion regulation, together with the ventrolateral and dorsomedial PFC that regulate activation in the amygdala and hypothalamus via the ACC (Berboth & Morawetz, 2021; Lopez, Denny, & Fagundes, 2018). Our findings indicate that CM could impact emotion regulation abilities particularly by affecting the connectivity of the amygdala to the dlPFC within this network. Notably, within individuals with an acute MDD, CM severity explained over 9% of the variance in amygdala-frontal connectivity indicating a moderate to large effect. Considering that small effect sizes are rather common in the domain of psychiatric neuroimaging (Jia et al., 2018; Schmaal et al., 2017, 2016), this observation points to the potential relevance of CM for this particular subgroup.

Notably, the effect of continuous CM on amygdala-frontal connectivity was not found when applying a more conservative motion thresholding. This finding suggests that the identification of maltreatment effects on resting-state functional connectivity is not robust across different preprocessing/quality assurance pipelines which could explain the heterogeneity in previous findings. Motion thresholding always aims at finding the right balance between removing too little artifactual and too much true variance in the data. The researcher degrees of freedoms created by the multitude of different settings possible in neuroimaging analysis may be one major reason for low replicability that has been observed in this domain (Marek et al., 2022). Unfortunately, there is no broad consensus regarding the optimal preprocessing steps and parameters. The utilization of largely automated preprocessing pipelines such as fMRIPrep can reduce the researcher degrees of freedom and could possibly contribute to higher replicability of findings (Esteban et al., 2019).

While interactions of CM with comorbidity profiles and with sex were not significant, we observed descriptively larger effect sizes in an MDD group with comorbid anxiety disorders and in female as compared to male MDD patients, thus tentatively pointing to the potential relevance of these additional subgroups. Importantly, the relevance of the acute MDD group was not driven by a higher load of comorbidities or differences in age and sex characteristics as compared to the remitted MDD sample.

Null-findings yielded by analyses with alternate CM operationalizations (i.e. categorical) raise the question how robust CM effects on resting-state connectivity are (whereas depending on specific operationalizations). Findings could tentatively point to a higher sensitivity of continuous operationalizations of maltreatment severity to identify functional connectivity correlates of CM. This is in line with recent findings suggesting superior psychometric qualities of continuous operationalizations (Goltermann et al., 2021).

Of note, even though amygdala-frontal connectivity alterations are generally in line with previous studies on CM, there is no consensus on the direction of the effects (Birn et al., 2014; Kaiser et al., 2018; van der Werff et al., 2013). While we observed stronger positive connectivity between the amygdala and frontal regions with increasing CM severity, the above-cited studies reported increased anticorrelation (Birn et al., 2014), decreased positive correlation (van der Werff et al., 2013) and a mixture of both (Kaiser et al., 2018) which can be summarized as a negative relationship of CM with connectivity as opposed to our findings.

In contrast, we did not find any evidence for altered amygdala connectivity as a function of CM severity in healthy subjects. Instead, weaker evidence was observed for an association with increased positive connectivity of the scACC with temporal and frontal regions, and with increased negative connectivity of the hippocampus with parts of the cerebellum. However, these effects did not survive multiple comparison corrections. No effects involving any other subdivisions of the ACC were found.

Furthermore, no evidence was found for a CM effect involving the resting-state connectivity of the subgenual portion of the ACC, contrasting findings of previous studies on adolescents (Herringa et al., 2013; Hoffmann et al., 2018) and adult samples (Thomason et al., 2015). Similarly, only very limited evidence for CM effects of altered pgACC connectivity was found. Merely in a secondary subgroup analysis, comparing severe CM with no CM within the MDD sample CM was associated with higher connectivity between the pgACC and clusters located in the supramarginal and superior temporal gyrus tentatively pointing to differential connectivity patterns for this population.

The significant and non-significant findings in the subgroup analyses of MDD and HC individuals could suggest differing effects depending on depressive psychopathology. However, the non-significant interaction effect between CM and diagnosis opposed to the differential findings obtained from HC and MDD samples in separate subgroup analyses seem to contradict each other. This could be due to the small effect sizes that were obtained within groups, potentially leading to insufficient slope differences to produce a significant interaction effect. In addition, the validity of investigating a CM main effect within the pooled samples, as well as the validity of the interaction effect is somewhat limited due to considerable heterogeneity in variance and distribution of CM reports across groups.

The effect sizes obtained in the current study, as well as the conducted power analysis suggest that even the largest previous studies (e.g. *N* = 88 in the study by van der Werff et al., 2013) could have been underpowered to detect relevant maltreatment-related connectivity alterations, potentially accounting for the large heterogeneity in previous findings. In contrast, our study should have had sufficient statistical power to detect effect sizes reported in previous studies. Overall, out of 30 primary statistical tests on different ROIs and subsamples, only one analysis of ROI connectivity produced robust significant results after correcting for multiple tests (right amygdala ROI in MDD). Even when applying a more lenient significance threshold, only two additional ROIs showed altered connectivity associated with CM (scACC and Hippocampus in HCs), again pointing to rather subtle CM effects.

An important limitation of the current study are the retrospective self-reports that were used to measure CM which rely on the ability of participants to accurately recall and report upon their childhood experiences despite potential cognitive depressive bias (Hardt & Rutter, 2004). However, recent findings of our work group indicate that retrospective self-reports using the CTQ are very stable over time and do not vary greatly as a function of changes in depressive symptoms (Goltermann et al., 2021). Another limiting factor of the reported findings arises from the specific methodology and sample used. While we aimed to account for a variety of different CM operationalizations in order to preclude that null-findings arise only as a result of a specific analytical approach, there still remains a variety of other methods that have previously been used, such as different preprocessing strategies or analysis approaches (e.g. investigation of ROI-to-ROI, voxel-to-voxel or dynamic resting-state connectivity, or regional homogeneity), as well as alternate cross-site harmonization approaches (e.g. NeuroCombat; Fortin et al., 2018), that might be more sensitive to detect neurobiological effects of maltreatment. Further, the neurobiological and clinical consequences of CM could heavily depend on the exact type and timing of the CM experiences, as indicated by an increasing number of studies (Gee et al., 2013; Herzog et al., 2020; Humphreys et al., 2019; Pechtel, Lyons-Ruth, Anderson, & Teicher, 2014; Zhu et al., 2019), as well as the subjective perception of stressors (Slavich & Shields, 2018). As this information is not captured by the CTQ this constitutes an additional important limitation of the current study. Another reason for heterogeneous and null-findings within the MDD sample could be the inherent heterogeneity of the disorder itself. Major depression is a disorder with various clinical manifestations and subtypes that could have differential neurobiological underpinnings (Drysdale et al., 2017; Fried & Nesse, 2015; Liu, Li, Li, Ren, & Ma, 2021; Milaneschi, Lamers, Berk, & Penninx, 2020; Vassilopoulou et al., 2013). It would be conceivable that CM-related connectivity changes vary across these subtypes of depression and future research may profit from thorough stratification. In summary, we present evidence for only subtle fronto-limbic connectivity alterations as a neural correlate of self-reported CM in depression. Experiences of maltreatment may play a particular role for the functional connectivity between the amygdala and the dlPFC during the presence of acute depressive symptoms. However, our findings illustrate the need for large-scale replications of previous studies with small sample sizes in the domain of psychiatric neuroimaging research in order to secure and reevaluate our knowledge in this domain. In the face of the enormous heterogeneity in localization and direction of neurobiological effects in previous studies, such large-scale replications are crucial to differentiate true and meaningful effects from false-positive findings. Future studies should further investigate the reliability as well as mechanistic nature of this relationship and whether this knowledge can be translated into personalized interventions to improve clinical treatment of affective disorders.

## Supplementary material

For supplementary material accompanying this paper visit https://doi.org/10.1017/S0033291722001623. click here to view supplementary material
