# Supplementary material for: Resting-state functional connectivity patterns associated with childhood maltreatment in a large bicentric cohort of adults with and without major depression
Source: Psychol Med. 2022 Jun 27;53(10):4720–31. doi: 10.1017/S0033291722001623 (PMC10388325; doi:10.1017/S0033291722001623)
Supplement: Supplementary file 1 [file S0033291722001623sup001.docx]

**Supplements**

**Supplementary Materials**

*Categorical operationalization of childhood maltreatment*

Following the procedure described by Walker et al. all participants exceeding the cutoffs in one of the five subscales of the childhood trauma questionnaire (CTQ) were categorized as maltreated. The validated cutoffs for each maltreatment subscale are: physical abuse ≥ 8, physical neglect ≥ 8, emotional neglect ≥ 15, emotional abuse ≥ 10, sexual abuse ≥ 8 (Walker et al., 1999). All other participants (not exceeding any of the subscale cutoffs) were categorized as non-maltreated.

*Calculation of medication index*

A medication index was calculated expressing the level of current psychiatric medication in terms of dosage of the combined current medication. Assessment of acute medication was done based on interviews conducted by trained personnel. Following the interview, all recorded medication was then classified by the corresponding active ingredients and medication categories (e.g. selective serotonin reuptake inhibitors, benzodiazepines; somatic medication was grouped in categories as contraceptives, thyroid or diabetes medication). Subsequently, all active ingredients of psychiatric medication were coded ‘0’, ‘1’ or ‘2’ based on established dosage-dependent cutoffs (Benkert & Hippius, 2015; Reynolds, 2008). These scores were then added together across all psychiatric medication to results into the final medication index. This procedure has been used in numerous previous publications (Redlich et al., 2014, 2015).

*Resting-state functional MRI aquisition*

Prescan normalize functionality was added to the scanning protocol during recruitment to correct for non-uniform receiver coil profiles prior to imaging. Only subjects for which a prescan normalize was applied were used in all analyses.

Resting state functional images were acquired in ascending order over a total duration of eight minutes (eyes closed) using a T2* weighted echo planar imaging sequence (EPI), which is sensitive to the blood oxygen level dependent contrast (BOLD; matrix = 64 x64, slices = 33, FOV = 210, voxel size = 3.3 x 3.3 x 3.8 mm, slice thickness = 3.8mm, slice gap = 10%, TE = 30 ms, TR = 2.0 s, flip angle = 90°). All slices covered the whole brain and were positioned transaxially parallel to the anterior-posterior commissural line with a tilted angle of 20°.

*Preprocessing pipeline and quality assurance protocol*

Resting-state preprocessing was done with the CONN (v18b) MATLAB toolbox using the default volume-based MNI preprocessing pipeline (<https://web.conn-toolbox.org/>) (Whitfield-Gabrieli & Nieto-Castanon, 2012). First, a series of preprocessing steps was applied to the functional and structural images that included a functional realignment and unwarp, a slice-timing correction, an ART-based outlier identification (acquisitions with framewise displacement above 0.9mm or global BOLD signal changes above 5 standard deviations were flagged as outliers), a direct segmentation and normalization of the functional and structural images, as well as a final functional smoothing with an 8mm FWHM kernel. Importantly, varying framewise displacement (FD) calculations have been reported in the literature. Thus, thresholds for identifying individual scans as outliers may differ across FD calculations and are not directly comparable. Here, we rely upon the composite motion estimate as implemented in the CONN toolbox, using default settings. Second, CONN’s denoising step with default parameters was applied to regress out potential noise artefacts in the functional data based on an anatomical component-based noise correction procedure (aCompCor). Controlling for noise artefacts is done by regressing out noise components from cerebral white matter and cerebrospinal areas (5 PCA components), estimated subject-motion parameters (12 parameters including 6 motion parameters and their associated first-order derivatives), as well as identified outlier scans or scrubbing. Subsequently, default temporal band-pass filtering was applied to remove low frequencies under 0.008 Hz and high frequencies above 0.09 Hz.

Finally, individual voxel-to-voxel connectivity distribution histograms (after denoising) were visually checked for irregularities as skewed distributions that could indicate artificial hyperconnectivity. These connectivities were then correlated with quality check parameters in order to assess if higher functional connectivities were associated with (and potentially produced by) mean and maximum motion, mean and maximum global signal change or the number of valid scans after scrubbing (so-called FC-QC correlations) (Ciric et al., 2017). Visual inspection of the distribution of FC-QC correlations did not yield any irregularities neither in the pooled sample nor in HC or MDD samples separately.

Motion metrics across diagnostic and maltreatment groups are presented in Table S5. To check that subjects with high motion did not drive the significance of clusters, we repeated all significant analyses while excluding 9 subjects that did not reach a minimum of 5 minutes valid resting state data (less than 150 non-scrubbed frames) (Satterthwaite et al., 2013).

In addition, we first repeated the ART outlier detection with the “conservative” setting in CONN (FD above 0.5mm and BOLD global signal change above 3 s.d.) and then repeated the denoising and first-level as well as previously significant second-level analyses. The conservative threshold resulted in further 93 subjects that needed to be excluded due to less than 5 minutes of non-scrubbed scans.

*Sample exclusion criteria*

Exclusion criteria were any history of neurological (e.g., concussion, stroke, tumor, neuro-inflammatory diseases) and medical (e.g., cancer, chronic inflammatory or autoimmune diseases, heart diseases, diabetes mellitus, infections) conditions and any further MRI contraindications. Participants under the current influence of medication including benzodiazepines, z-drugs or stimulants were further excluded, as well as patients with an anorexia nervosa or substance dependence comorbidity, and non-Caucasian participants. The latter were excluded because the utilized cohort was originally recruitment for the purpose of genetic and neuroimaging analyses, thus aiming to create a genetically homogeneous sample.

*Power analysis*

We calculated the required minimum effect size necessary to obtain a statistical power of .95 (sensitivity), given alpha .001 and the available sample sizes of samples that were analyzed. The power analysis was conducted using g*Power (Faul, Erdfelder, Lang, & Buchner, 2007) testing for single regression coefficient in a linear multiple regression model. Input parameters were two-tailed testing, alpha .001, power .95, respective sample size, and respective number of predictors (four predictors in subsamples and five predictors in pooled sample after adding the diagnosis control variable). The resulting effect size f² was converted to η²_partial_ based on established formulas (Cohen, 1988; Rosenthal & Rosnow, 2008).

**Supplementary Results**

*Maltreatment subtype-specific effects on connectivity*

It has been frequently discussed whether different forms of maltreatment can be lumped together or need to be investigated separately with regard to their specificity for neurobiological and clinical consequences (Smith & Pollak, 2021). To follow-up on our main effect of general maltreatment severity on the connectivity between the right amygdala seed ROI and the dorsolateral prefrontal cortex (dlPFC) cluster we probed maltreatment subtype effects (i.e. emotional and physical neglect, as well as emotional, physical and sexual abuse) via the CTQ subscales in an exploratory fashion. This analysis revealed that descriptively, emotional abuse and physical neglect had the largest and sexual abuse the smallest effect size on amygdala-dorsolateral prefrontal cortex dlPFC connectivity. However, there was large overlap between effect size confidence intervals of all subscales. The comparison of effect sizes of different maltreatment subtypes on the amygdala-dlPFC connectivity are shown in Table S4. See also Table S9 showing intercorrelations between subscales.

When testing maltreatment subtype effects in CONN with original significance thresholds corrected for multiple comparisons, only emotional abuse and physical neglect yielded significant effects of altered connectivity of the right amygdala with a dlPFC cluster largely overlapping with the result obtained when testing a CTQ sum effect (emotional abuse: k=143, x21, y45, z48, p_FDR_=.003, η²_partial_=.041, bias corrected and accelerated (BCa) 95%-CI: [.016, .082]; physical neglect: k=123, x36, y27, z54, p_FDR_=.010, η²_partial_=.043, BCa 95%-CI: [.020, .077]. Emotional abuse was additionally associated with higher connectivity between the right amygdala and a cluster within the middle cingulate cortex (k=92, x0, y-36, z48, p_FDR_=.012, η²_partial_=.033, BCa 95%-CI: [.013, .061]).

Differences in subscale findings should be interpreted with caution due to strong correlations between subscales, as well as lower psychometric qualities (particularly physical neglect; Wingenfeld et al., 2011) and extreme skewness of some of the subscales (particularly sexual abuse).

*The role of individuals’ sex*

In order to investigate the potential role of participants’ biological sex for maltreatment-associated connectivity differences we followed up on the main finding by testing for an interaction effect between CTQ sum and sex on the extracted cluster connectivities between the right amygdala seed ROI and the frontal cluster within the MDD sample. This yielded a non-significant CTQ X sex interaction (p=.331). Investigating extracted cluster connectivities of male and female participants separately revealed larger effects of CTQ sum on amygdala-dlPFC connectivity within female participants (n=414, η²_partial_=.065, BCa 95%-CI: [.030, .114]), as compared to male participants for which the CTQ sum effect was not significant (n=210, η²_partial_=.017, BCa 95%-CI: [-.000, .079]). Investigating female and male participants separately within the CONN software yielded a significant effect of CTQ sum on right amygdala connectivity to a frontal cluster (similar to the main finding) in female MDD participants only (k=83, x24, y42, z48, p_FDR_=.042, η²_partial_=.051, BCa 95%-CI: [.019, .099]), with no significant clusters emerging for the male MDD sample. Testing for a CTQ sum X sex interaction in CONN yielded no significant clusters for the right amygdala seed ROI when applying the combined uncorrected voxel threshold and the FDR-corrected cluster size threshold.

*The role of comorbidities*

The utilized sample included a large subgroup of MDD patients with various comorbid disorders (see Table S2). As previous research suggests that various mental disorders other than MDD are also associated with experiences of CM (Lippard & Nemeroff, 2020; Struck et al., 2020), as well as with alterations in resting-state connectivity (Doucet et al., 2020; Oathes, Patenaude, Schatzberg, & Etkin, 2015) we conducted additional analyses stratifying the MDD group for comorbidities to follow-up on our main finding with CTQ sum association with right amygdala connectivity. To this end, MDD subgroups with lifetime comorbid anxiety disorders (n=192) or any other lifetime comorbidity (n=60) were investigated separately and compared to a MDD subgroup without any comorbid disorders (n=372) in order to investigate the role of these comorbidity profiles on the connectivity effect.

Investigating the connectivity of the right amygdala seed in these groups separately within the CONN software using original significance thresholds (including correction for multiple tests) yielded no clusters with a significant CTQ sum main effect in either subgroup, presumably due to lower statistical power within these subsamples. Further, no clusters emerged with a significant interaction effect between CTQ sum and comorbidity groups.

In order to further investigate potential differences between these groups we calculated effect size estimates for both subsamples separately for the individual extracted right amygdala-dlPFC cluster connectivities where we located our main effect of CTQ sum within the entire MDD sample. The effect size of the CTQ sum main effect on extracted cluster connectivities between the right amygdala and the dlPFC cluster was descriptively larger within the MDD group with anxiety comorbidities (η²_partial_=.097, BCa 95%-CI: [.030, .179]) as compared to the MDD group without any comorbidities (η²_partial_=.031, BCa 95%-CI: [.005, .074]). Within the MDD group with a comorbidity other than an anxiety disorder the main effect of CTQ sum on extracted cluster connectivities was non-significant (η²_partial_=.004, BCa 95%-CI: [.000, .120]). The difference in effect sizes between groups was not statistically significant as indicated by a non-significant interaction term between CTQ sum and MDD comorbidity groups (p=.333). For the CTQ sum effect on cluster connectivities stratified by comorbidity groups see Figure S3 A.

Investigating the main effect of comorbidity group on extracted cluster connectivities (entering a factor with all three comorbidity groups as a predictor, controlling for age, sex, and scanner specifications) yielded a significant effect (p<.001, η²_partial_=.025). Follow-up pairwise comparisons revealed that this main effect was driven by higher amygdala-dlPFC connectivities in the MDD group with other comorbidities as compared to both – the group without comorbidities (p<.001) and the group with anxiety comorbidities (p=.029). The latter two did not significantly differ (p=.089) (see Figure S3 B).

*Sensitivity analyses*

We conducted several sensitivity analyses in order to investigate if the observed maltreatment-associated functional connectivity of the right amygdala in MDD (main finding) was driven by certain comorbidities or medication within the MDD samples. To this aim in a first step we excluded all participants with a comorbid eating disorder, substance abuse disorder or psychotic disorder, as these diagnoses have been specifically associated with altered brain structure and function (Blest-Hopley, Giampietro, & Bhattacharyya, 2018; Frank, 2019; McAdams & Smith, 2015; Tanabe, Regner, Sakai, Martinez, & Gowin, 2019; Wolf et al., 2020) and could thus drive the observed connectivity effects of maltreatment. An exclusion of these participants (overall n=70 excluded) yielded highly comparable results for the main effect of CTQ sum on the right amygdala connectivity (altered connectivity with cluster in the dlPFC, k=152, x21, y39, z48, p_FDR_=.003, η²_partial_=.046, BCa 95%-CI: [.017, .083]).

In order to control for the impact of medication on the observed connectivity effect of the right amygdala we added the medication index variable as an additional covariate to the model in a follow-up analysis. When adding medication index to the model in the seed-to-voxel CONN analysis of the right amygdala seed while applying original significance thresholds also yielded highly similar results as compared to the original model in regard to the dlPFC cluster (k=127, p_FDR_=.009, η²_partial_=.039, BCa 95%-CI: [.013, .076], peak voxel coordinates: x21, y45, z48), as well as the middle cingulate cluster (k=76, p_FDR_=.044, η²_partial_=.030, BCa 95%-CI: [.010, .062], peak voxel coordinates: x0, y-36, z48).

*Characteristics of acute and lifetime MDD samples*

In order to follow up on the observed differences between the lifetime MDD sample and the acute MDD subsample we compared acute and remitted MDD participant groups with regard to demographic and clinical characteristics. Mean age (mean difference: -2.74, BCa 95%-CI [-2.301, 1.807], p=.789), as well as proportion of female participants (χ^2^(1,N=624)=1.114, p=.291) did not differ across acute and remitted MDD samples.

Both groups did also not differ in the presence of comorbidities (any lifetime comorbidity: χ^2^(1,N=624)=.524, p=.469; number of comorbidities: χ^2^(4,N=624)=1.064, p=.900; comorbid lifetime anxiety disorder: χ^2^(1,N=624)=.047, p=.829). Further, groups did not differ in presence or absence of childhood maltreatment based on the categorical CTQ cutoffs (χ^2^(1,N=624)=.214, p=.644). However, the acute MDD group had on average higher CTQ sum scores (mean difference: 2.642, BCa 95%-CI [0.159, 5.191], p=.045) and more previous hospitalizations (mean difference: 0.531, BCa 95%-CI [0.204, 0.877], p=.045) as compared to the remitted MDD group.

*Alternative maltreatment operationalizations*

Several secondary analyses were conducted in order to explore if findings from our main analysis would be robust across different CM operationalizations. Firstly, the cutoff-based categorization approach was applied grouping the sample into individuals with and without CM (CM vs. non-CM). Secondly, we compared tail percentage CTQ groups, i.e. individuals that reported severe CM (10% highest CTQ sum points) were compared to individuals with very low reports of CM (10% lowest CTQ sum points), in order to explore if CM effects could be found particularly in groups with high CM loads. No tail percentage group comparisons (severe CM vs. non-CM) were conducted within the acute MDD sample due to insufficient cell sizes. No significant main effects of CM on resting-state functional connectivity were found for any operationalizations when pooling HC and MDD samples together in one model. Results of the subgroup analyses using different CM operationalizations are presented in Table S6.

*Alternative multiple comparison correction*

To correct for multiple comparisons, we conducted cluster-level multiple comparison correction using a voxel-wise threshold of p<.001 and a cluster-level FDR-corrected threshold of p<.05 based on Gaussian Random Field theory (Worsley et al. 1996). Since Eklund et al. (2016) have shown that type-I errors can be inflated in some scenarios, we repeated all analyses initially showing a significant cluster result now using a Threshold-Free Cluster Enhancement (TFCE) approach (Smith & Nichols, 2009) in order to control the family-wise error (FWE). The approach has the additional benefit of avoiding user-defined voxel- or cluster-level thresholds and takes the empirical smoothness of the data into account.

Recomputing the seed-to-voxel analysis using the right Amygdala as seed region still shows a significant connectivity cluster with the dlPFC, similar to the results we report using Random Field FDR-cluster correction, although the TFCE-corrected cluster is smaller in size (peak-coordinates: +24 +39 +45, size=24, TFCE=430.87, peak p-FWE=.046).

**References**

Benkert, O., & Hippius, H. (Eds.). (2015). *Kompendium der Psychiatrischen Pharmakotherapie* (10th ed.). Berlin Heidelberg: Springer Verlag.

Blest-Hopley, G., Giampietro, V., & Bhattacharyya, S. (2018). Residual effects of cannabis use in adolescent and adult brains — A meta-analysis of fMRI studies. *Neuroscience and Biobehavioral Reviews*, *88*, 26–41. https://doi.org/10.1016/j.neubiorev.2018.03.008

Ciric, R., Wolf, D. H., Power, J. D., Roalf, D. R., Baum, G. L., Ruparel, K., … Satterthwaite, T. D. (2017). Benchmarking of participant-level confound regression strategies for the control of motion artifact in studies of functional connectivity. *NeuroImage*, *154*, 174–187. https://doi.org/10.1016/j.neuroimage.2017.03.020

Cohen, J. (1988). *Statistical Power Analysis for the Behavioral Sciences* (2nd ed.). New York, NY: Academic Press.

Doucet, G. E., Janiri, D., Howard, R., O’Brien, M., Andrews-Hanna, J. R., & Frangou, S. (2020). Transdiagnostic and disease-specific abnormalities in the default-mode network hubs in psychiatric disorders: A meta-analysis of resting-state functional imaging studies. *European Psychiatry*, *63*(1), 1–7. https://doi.org/10.1192/j.eurpsy.2020.57

Faul, F., Erdfelder, E., Lang, A.-G., & Buchner, A. (2007). G*Power 3: A flexible statistical power analysis program for the social, behavioral, and biomedical sciences. *Behavior Research Methods*, *39*(2), 175–191.

Frank, G. K. W. (2019). Neuroimaging and eating disorders. *Current Opinion in Psychiatry*, *32*(6), 478–483. https://doi.org/10.1097/YCO.0000000000000544

Lippard, E. T. C., & Nemeroff, C. B. (2020). The Devastating Clinical Consequences of Child Abuse and Neglect: Increased Disease Vulnerability and Poor Treatment Response in Mood Disorders. *The American Journal of Psychiatry*, *177*(1), 20–36. https://doi.org/10.1176/appi.ajp.2019.19010020

McAdams, C., & Smith, W. (2015). Neural correlates of eating disorders: translational potential. *Neuroscience and Neuroeconomics*, *4*, 35–49. https://doi.org/10.2147/nan.s76699

Oathes, D. J., Patenaude, B., Schatzberg, A. F., & Etkin, A. (2015). Neurobiological signatures of anxiety and depression in resting-state functional magnetic resonance imaging. *Biological Psychiatry*, *77*(4), 385–393. https://doi.org/10.1016/j.biopsych.2014.08.006

Redlich, R., Almeida, J. R., Grotegerd, D., Opel, N., Kugel, H., Heindel, W., … Dannlowski, U. (2014). Brain Morphometric Biomarkers Distinguishing Unipolar and Bipolar Depression: A Voxel-Based Morphometry-Pattern Classification Approach. *JAMA Psychiatry*, *71*(11), 1222–1230. https://doi.org/10.1001/jamapsychiatry.2014.1100

Redlich, R., Dohm, K., Grotegerd, D., Opel, N., Zwitserlood, P., Heindel, W., … Dannlowski, U. (2015). Reward Processing in Unipolar and Bipolar Depression: A Functional MRI Study. *Neuropsychopharmacology : Official Publication of the American College of Neuropsychopharmacology*, *40*(11), 2623–2631. https://doi.org/10.1038/npp.2015.110

Reynolds, C. R. (2008). Physicians’ Desk Reference. In *Encyclopedia of Special Education*. Vol Hoboken, JK, USA: John Wiley Sons, Inc.

Rolls, E. T., Huang, C. C., Lin, C. P., Feng, J., & Joliot, M. (2020). Automated anatomical labelling atlas 3. *NeuroImage*, *206*(August 2019), 116189. https://doi.org/10.1016/j.neuroimage.2019.116189

Rosenthal, R., & Rosnow, R. L. (2008). *Essentials of Behavioral Research* (3rd ed.; E. Barrosse, ed.). New York: McGraw-Hill.

Satterthwaite, T. D., Elliott, M. A., Gerraty, R. T., Ruparel, K., Loughead, J., Calkins, M. E., … Wolf, D. H. (2013). An improved framework for confound regression and filtering for control of motion artifact in the preprocessing of resting-state functional connectivity data. *NeuroImage*, *64*(1), 240–256. https://doi.org/10.1016/j.neuroimage.2012.08.052

Smith, K. E., & Pollak, S. D. (2021). Rethinking Concepts and Categories for Understanding the Neurodevelopmental Effects of Childhood Adversity. *Perspectives on Psychological Science*, *16*(1), 67–93. https://doi.org/10.1177/1745691620920725

Struck, N., Krug, A., Yuksel, D., Stein, F., Schmitt, S., Meller, T., … Brakemeier, E. L. (2020). Childhood maltreatment and adult mental disorders – the prevalence of different types of maltreatment and associations with age of onset and severity of symptoms. *Psychiatry Research*, *293*(August), 113398. https://doi.org/10.1016/j.psychres.2020.113398

Tanabe, J., Regner, M., Sakai, J., Martinez, D., & Gowin, J. (2019). Neuroimaging reward, craving, learning, and cognitive control in substance use disorders: Review and implications for treatment. *British Journal of Radiology*, *92*(1101). https://doi.org/10.1259/bjr.20180942

Walker, E. A., Gelfand, A., Katon, W. J., Koss, M. P., Korff, M. Von, Bernstein, D., & Russo, J. (1999). Adult Health Status of Women with Histories of Childhood Abuse and Neglect. *The American Journal of Medicine*, *107*, 332–339.

Whitfield-Gabrieli, S., & Nieto-Castanon, A. (2012). Conn: A Functional Connectivity Toolbox for Correlated and Anticorrelated Brain Networks. *Brain Connectivity*, *2*(3), 125–141. https://doi.org/10.1089/brain.2012.0073

Wingenfeld, K., Schäfer, I., Terfehr, K., Grabski, H., Driessen, M., Grabe, H., … Spitzer, C. (2011). The Reliable, Valid and Economic Assessment of Early Traumatization: First Psychometric Characteristics of the German Version of the Adverse Childhood Experiences Questionnaire (ACE). *Psychotherapie Psychosomatik Medizinische Psychologie*, *61*(1), e10–e14. https://doi.org/10.1055/s-0030-1263161

Wolf, R. C., Hildebrandt, V., Schmitgen, M. M., Pycha, R., Kirchler, E., MacIna, C., … Huber, M. (2020). Aberrant Gray Matter Volume and Cortical Surface in Paranoid-Type Delusional Disorder. *Neuropsychobiology*, *79*(4–5), 335–344. https://doi.org/10.1159/000505601

Table S1. Demographic and clinical characteristics of healthy control and lifetime major depressive disorder samples.

|  | HC | |  | MDD | | p-value |
| --- | --- | --- | --- | --- | --- | --- |
|  | mean (SD) | count |  | mean (SD) | count |  |
| Age | 35.75 (13.02) | - |  | 36.64 (13.10) | - | .215 |
| Sex f/m | - | 453/248 |  | - | 414/210 | .510 |
| CTQ sum | 32.99 (8.93) | - |  | 45.36 (15.96) | - | <.001 |
| CM yes/no | - | 217/484 |  | - | 427/197 | <.001 |
| BDI sum | 4.50 (4.37) | - |  | 17.32 (10.90) | - | <.001 |
| GAF | 89.30 (13.56) | - |  | 63.71 (15.77) | - | <.001 |
| Number of psychiatric hospitalizations | - | - |  | 1.53 (2.02) | - | - |
| Years of education | 13.93 (2.68) | - |  | 13.16 (2.74) | - | <.001 |

*Note.* Statistical tests of group differences were done via t-tests for continuous and χ²-tests for categorical variables. As CTQ sum, BDI sum and GAF all produced significant Levene’s tests, t-tests for unequal variance were conducted. HC, healthy controls; MDD, major depressive disorder; f, female; m, male; CTQ, Childhood Trauma Questionnaire; CM, childhood maltreatment; BDI, Beck Depression Inventory; GAF, Global Assessment of Functioning Scale.

Table S2. Lifetime comorbidities, incidences of childhood maltreatment subtypes, and medication of the sample with a lifetime depressive disorder diagnosis (N=624).

|  | N | % |
| --- | --- | --- |
| *Comorbid diagnoses* |  |  |
| Anxiety disorders | 192 | 30.8 |
| Eating disorders | 36 | 5.8 |
| Substance disorders | 30 | 4.8 |
| Dysthymic disorder | 29 | 4.6 |
| Somatoforme disorders | 17 | 2.7 |
| Psychotic disorders | 6 | 1 |
| *Number of comorbidities* |  |  |
| 0 | 372 | 59.6 |
| 1 | 159 | 25.5 |
| 2 | 64 | 10.3 |
| 3 | 22 | 3.5 |
| 4 | 7 | 1.1 |

*Note.* Lifetime comorbidities were derived from the structured clinical interview for DSM-IV (SCID). Lifetime anxiety disorders implicated specific phobias, social anxiety disorder, panic and agoraphobic disorders, generalized anxiety disorder, posttraumatic stress disorder, obsessive-compulsive disorder, anxiety disorder not otherwise specified (NOS) and adjustment disorder with mixed anxiety and depressed mood. Eating disorders implicated bulimia nervosa and eating disorder NOS. Among the category of substance disorders were cannabis abuse and alcohol abuse, as well as abuse of other substances. The group of participants with somatoforme disorders entailed somatoforme pain disorder, somatization disorder or somatization disorder NOS. The category of psychotic disorders included delusional disorder, brief psychotic disorder and psychotic disorder NOS.

Table S3. Categorical prevalence of childhood maltreatment subtypes across healthy control and major depressive disorder participants.

|  | HC (n=701) | |  | Lifetime MDD (n=624 | |
| --- | --- | --- | --- | --- | --- |
|  | N | % |  | N | % |
| *Prevalence of categorical childhood maltreatment subtypes* |  |  |  |  |  |
| Emotional abuse | 99 | 14.1 |  | 317 | 50.8 |
| Physical abuse | 61 | 8.7 |  | 144 | 23.1 |
| Sexual abuse | 29 | 4.1 |  | 109 | 17.5 |
| Emotional neglect | 61 | 8.7 |  | 244 | 39.1 |
| Physical neglect | 138 | 19.7 |  | 274 | 43.9 |
| *Number of categorical maltreatment subtypes per person* |  |  |  |  |  |
| 0 | 484 | 69.0 |  | 197 | 31.6 |
| 1 | 127 | 18.1 |  | 129 | 20.7 |
| 2 | 40 | 5.7 |  | 88 | 14.1 |
| 3 | 26 | 3.7 |  | 92 | 14.7 |
| 4 | 17 | 2.4 |  | 83 | 13.3 |
| 5 | 7 | 1.0 |  | 35 | 5.6 |

*Note.* Prevalence of childhood maltreatment subtypes were based on categorical subscale cutoffs established by Walker et al. (1999). HC, healthy control; MDD, major depressive disorder.

Table S4. Association of maltreatment subtypes of the CTQ with extracted connectivities between right amygdala seed ROI and the dorsolateral prefrontal cluster that was found to significantly correlate with CTQ sum in the major depressive disorder sample (frontal cluster in Figure 1).

|  | p-value | Partial η² [BCa 95%-CI] |
| --- | --- | --- |
| CTQ sum | <.001 | .050 [.023, .085] |
| Emotional abuse | <.001 | .049 [.023, .086] |
| Physical abuse | <.001 | .018 [.003, .047] |
| Sexual abuse | .020 | .009 [<.001, .030] |
| Emotional neglect | <.001 | .035 [.012, .068] |
| Physical neglect | <.001 | .039 [.016, .073] |

*Note.* Main effects of CTQ subscale scores are shown on amygdala between right amygdala and frontal cluster identified in the main analysis. The original result for the CTQ sum score is presented on the top for reference. The same covariates were used in the statistical model as in the original analysis. Bias corrected and accelerated (BCa) 95% confidence intervals are shown due to skewed data of the predictors.

Table S5. Motion metrics during the resting-state functional neuroimaging sequence across diagnostic and maltreatment groups.

|  | Mean motion metrics | | |  | Difference statistics | |
| --- | --- | --- | --- | --- | --- | --- |
|  | Mean (SD) | Min | Max |  | Cohens d [95%-CI] | p-value |
| *Childhood maltreatment* |  |  |  |  |  |  |
| Non-maltreated (n=681) | 0.215 (0.093) | 0.065 | 0.727 |  | 0.232 [0.124, 0.340] | <.001 |
| Maltreated (n=644) | 0.241 (0.131) | 0.056 | 1.397 |  |  |  |
|  |  |  |  |  |  |  |
| *MDD diagnosis* |  |  |  |  |  |  |
| HC (n=701) | 0.215 (0.098) | 0.065 | 0.818 |  | 0.237 [0. 128, 0.345] | <.001 |
| Lifetime MDD (n=624) | 0.242 (0.128) | 0.056 | 1.397 |  |  |  |

*Note.* Motion metrics are presented in mm and intra-individually averaged across non-scrubbed frames. Min and max motion thus indicate maximum and minimum of individual averages within the samples. Categorical maltreatment is based on the subscale cutoffs established by Walker et al. (1999). MDD, major depressive disorder, HC, healthy control.

Table S6. Significant clusters with main effects of childhood maltreatment in ROI-based seed-to-voxel analyses for different CM operationalizations.

| Sample | N | CM operationalization | ROI | Main regions of significant cluster | k | peak-voxel coordinates | Direction of effect | p-FDR | p-FDR_Bonferroni_ | partial η² [95%-CI] |
| --- | --- | --- | --- | --- | --- | --- | --- | --- | --- | --- |
| HC | 701 | CM vs. non-CM | Hippocampus L | Cerebellum R | 67 | +27 -42 -42 | negative | .025 | .247 | .035 [.009, .060] |
|  |  |  |  |  |  |  |  |  |  |  |
| HC | 156 | severe CM vs. non-CM | scACC R | Medial superior frontal L+R | 89 | -6 +30 +54 | positive | .025 | .254 | .129 [.048, .241] |
|  |  |  |  |  |  |  |  |  |  |  |
| Lifetime MDD | 625 | CM vs. non-CM | Amygdala R | Middle cingulate L+R, Precuneus R | 85 | -3 -36 +51 | positive | .035 | .350 | .028 [.010, .055] |
|  |  |  |  |  |  |  |  |  |  |  |
| Lifetime MDD | 134 | severe CM vs. non-CM | pgACC L | Superior temporal R, Supramarginal R | 111 | +69 -36 +18 | negative | .008 | .083 | .169 [.067, .292] |
|  |  |  | pgACC L | Superior temporal L, Supramarginal L | 90 | -54 -42 +27 | negative | .011 | .114 | .165 [.071 , .283] |
|  |  |  | Hippocampus R | Postcentral R | 82 | +48 -27 +57 | positive | .017 | .171 | .125 [.042, .234] |

*Note.* FDR-corrected cluster-size p-values are presented with and without additional Bonferroni correction. The direction of effects indicates if the association between CTQ sum and seed-to-voxel connectivity is positive (higher positive or less negative connectivity with higher CTQ sum scores) or negative (higher negative or less positive connectivity with higher CTQ sum scores). Bootstrapped (N=1000 samples) bias-corrected and accelerated 95% confidence intervals are presented for all effect sizes. HC, healthy control; MDD, major depressive disorder; CTQ, childhood trauma questionnaire; CM, childhood maltreatment; sc, supracallosal; pg, pregenual; ACC, anterior cingulate cortex; L, left; R, right; CI, confidence interval.

Table S7. Overview of all primary and secondary analyses.

|  |  |  | Amygdala | |  | Hippocampus | |  | sgACC | |  | pgACC | |  | scACC | |
| --- | --- | --- | --- | --- | --- | --- | --- | --- | --- | --- | --- | --- | --- | --- | --- | --- |
| Sample | N | CM operationalization | left | right |  | left | right |  | left | right |  | left | right |  | left | right |
| ***Primary analyses*** |  |  |  |  |  |  |  |  |  |  |  |  |  |  |  |  |
| HC+MDD | 1325 | CTQ sum | ns | ns |  | ns | ns |  | ns | ns |  | ns | ns |  | ns | ns |
| HC | 701 | CTQ sum | ns | ns |  | 1 cluster | ns |  | ns | ns |  | ns | ns |  | ns | 3 clusters |
| MDD | 624 | CTQ sum | ns | 3 clusters |  | ns | ns |  | ns | ns |  | ns | ns |  | ns | ns |
| ***Secondary analyses*** |  |  |  |  |  |  |  |  |  |  |  |  |  |  |  |  |
| MDD acute | 264 | CTQ sum | ns | 1 cluster |  | ns | ns |  | ns | ns |  | ns | ns |  | ns | ns |
| HC+MDD | 1325 | CM vs. non-CM | ns | ns |  | ns | ns |  | ns | ns |  | ns | ns |  | ns | ns |
| HC+MDD | 320 | severe CM vs. non-CM | ns | ns |  | ns | ns |  | ns | ns |  | ns | ns |  | ns | ns |
| HC | 701 | CM vs. non-CM | ns | ns |  | 1 cluster | ns |  | ns | ns |  | ns | ns |  | ns | ns |
| HC | 156 | severe CM vs. non-CM | ns | ns |  | ns | ns |  | ns | ns |  | ns | ns |  | ns | 1 cluster |
| MDD | 624 | CM vs. non-CM | ns | 1 cluster |  | ns | ns |  | ns | ns |  | ns | ns |  | ns | ns |
| MDD | 134 | severe CM vs. non-CM | ns | ns |  | ns | 1 cluster |  | ns | ns |  | 2 clusters | ns |  | ns | ns |
| MDD acute | 264 | CM vs. non-CM | ns | ns |  | ns | ns |  | ns | ns |  | ns | ns |  | ns | ns |

*Note.* Number of significant clusters before applying Bonferroni-correction for multiple comparisons. CM versus non-CM analyses are using a cutoff-based categorization approach. Severe CM vs non-CM compare tail percentage CTQ groups, i.e. individuals that reported severe CM (10% highest CTQ sum points) were compared to individuals with very low reports of CM (10% lowest CTQ sum points). ns = non-significant already before Bonferroni correction, HC = healthy controls, MDD = major depressive disorder, CM = childhood maltreatment, sgACC = subgenual anterior cingulate cortex, pgACC = pregenual anterior cingulate cortex, scACC = supracallosal anterior cingulate cortex.

Table S8. Additional analyses controlling for confounding effects of medication, motion, and education (right amydala seed).

| Sample | Controlling for | N | k | peak-voxel coordinates | p-FDR | partial η² [95%-CI] |
| --- | --- | --- | --- | --- | --- | --- |
| MDD | age, sex, site, education | 622 | 92 | 0 -36 +48 | .037 | .033 [.012, .068] |
|  |  |  | 77 | +21 +42 +48 | .038 | .033 [.011, .067] |
| MDD | age, sex, site, mean motion | 624 | 124 | +21 +45 +48 | .010 | .038 [.014, .077] |
|  |  |  | 83 | 0 -36 +48 | .031 | .031 [.009, .066] |
| MDD | age, sex, site, medication | 624 | 127 | +21 +45 +48 | .009 | .039 [.015, .078] |
|  |  |  | 76 | 0 -36 +48 | .044 | .030 [.008, .062] |
| MDD | age, sex, site, mean motion, medication, education | 622 | 106 | 0 -36 +48 | .019 | .033 [.011, .069] |
|  |  |  | 89 | +21 +42 +48 | .020 | .033 [.011, .065] |
| MDD | age, sex, site, education, excluding subjects with less than 150 valid frames | 615 | 131 | +21 +42 +48 | .006 | .033 [.012, .065] |
|  |  |  | 87 | +24 +21 +54 | .022 | .034 [.012, .067] |
|  |  |  | 65 | 0 -36 +48 | .044 | .036 [.013, .067] |
| MDD | age, sex, site, mean motion, excluding subjects with less than 150 valid frames | 617 | 260 | +24 +12 +54 | <.001 | .053 [.024, .097] |
|  |  |  | 128 | 0 -36 +48 | .004 | .035 [.012, .069] |
|  |  |  | 83 | +24 -9 -21 | .017 | .039 [.015, .073] |
|  |  |  | 63 | -27 -15 -36 | .034 | .041 [.016, .075] |
| MDD | age, sex, site, medication, excluding subjects with less than 150 valid frames | 617 | 107 | +21 +45 +48 | .012 | .036 [.012, .072] |
|  |  |  | 103 | 0 -36 +48 | .012 | .034 [.012, .063] |
|  |  |  | 76 | +24 +12 +54 | .027 | .033 [.012, .067] |
| MDD | age, sex, site, mean motion, medication, education, excluding subjects with less than 150 valid frames | 615 | 142 | -3 -36 +48 | .004 | .037 [.013, .069] |

Table S8 continued.

| Sample | Controlling for | N | k | peak-voxel coordinates | p-FDR | partial η² [95%-CI] |
| --- | --- | --- | --- | --- | --- | --- |
| MDD | age, sex, site, mean motion, medication, education, excluding subjects with less than 150 valid frames |  | 86 | +21 +42 +48 | .018 | .035 [.013, .070] |
|  |  |  | 84 | +24 +21 +54 | .018 | .033 [.011, .068] |
| MDD | age, sex, site, conservative ART setting | 569 | 176 | -27 -15 -39 | <.001 | .062 [.026, .115] |
|  |  |  | 112 | +12 -39 +45 | .005 | .036 [.013, .076] |
|  |  |  | 88 | +24 -09 -21 | .011 | .043 [.017, .081] |

*Note.* MDD = major depressive disorder, FDR = false discovery rate, CI = confidence interval, ART = artifact detection tools.

Table S9. Spearman correlations between CTQ subscales

|  | EA | PA | SA | EN | PN |
| --- | --- | --- | --- | --- | --- |
| EA | 1 |  |  |  |  |
| PA | .509 [.466, .553] | 1 |  |  |  |
| SA | .354 [.312, .396] | .278 [.218, .335] | 1 |  |  |
| EN | .722 [.690, .751] | .473 [.429, .518] | .288 [.239, .333] | 1 |  |
| PN | .564 [.520, .606] | .398 [.352, .448] | .285 [.230, .334] | .674 [.639, .706] | 1 |

*Note*. Spearman rho is presented for associations between continuous CTQ subscales. Bootstrapped (N=1000 samples) bias-corrected and accelerated (BCa) 95% confidence intervals for rho are presented in brackets. EA, emotional abuse; PA, physical abuse; SA, sexual abuse; EN, emotional neglect; PN, physical neglect.


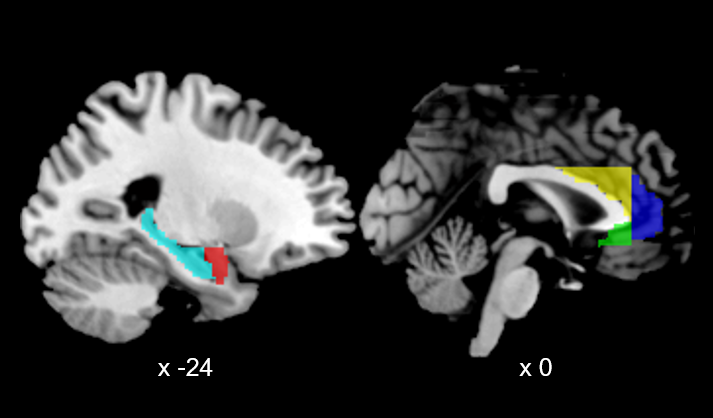


Figure S1. Regions of interest (ROIs) from the Automated Anatomical Labelling Atlas version 3 (Rolls, Huang, Lin, Feng, & Joliot, 2020) that were used as seed regions in the present study. All depicted ROIs were defined separately for the left and right hemisphere, thus resulting in a total of 10 ROIs. Slice positions are presented as x-coordinates in MNI-space. ROIs are depicted as follows: Cyan, hippocampus; red, amygdala; yellow, supracallosal anterior cingulate cortex (ACC); dark blue, pregenual ACC; green, subgenual ACC.


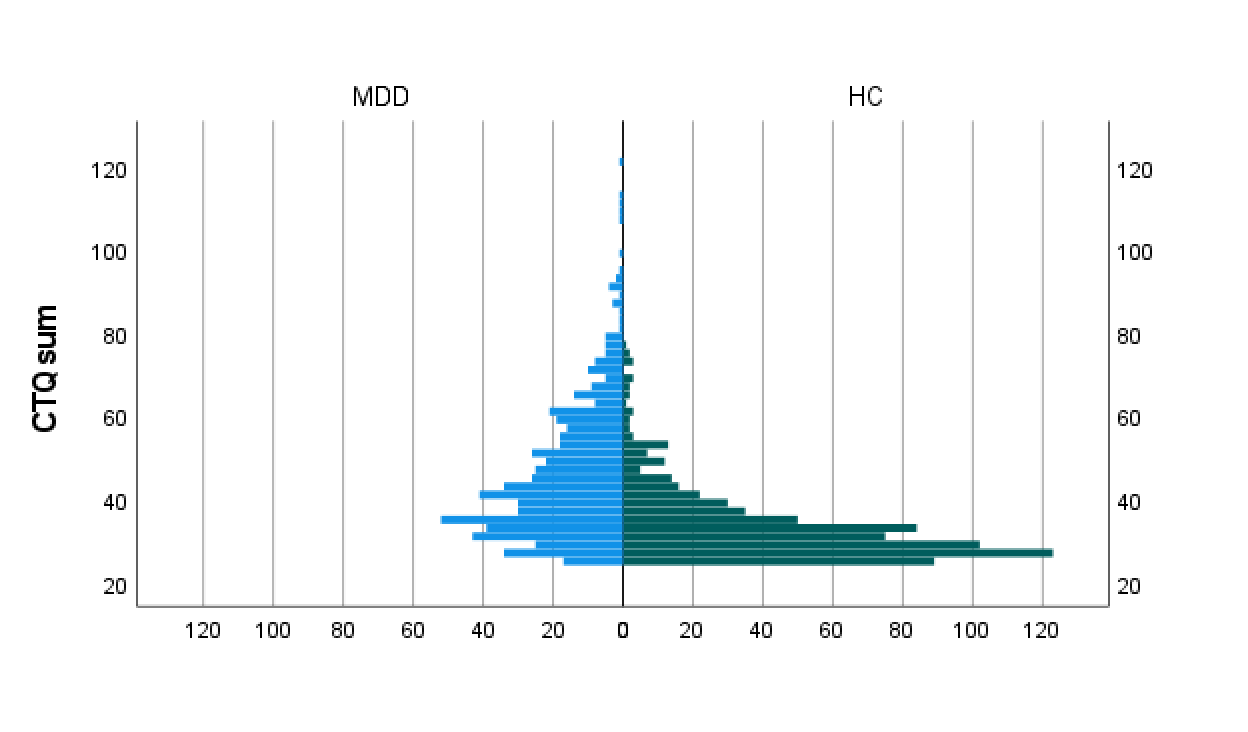


Figure S2. Histogram displaying the distribution of CTQ sum scores across MDD and HC subgroups. CTQ, childhood trauma questionnaire; MDD, major depressive disorder; HC, healthy controls.


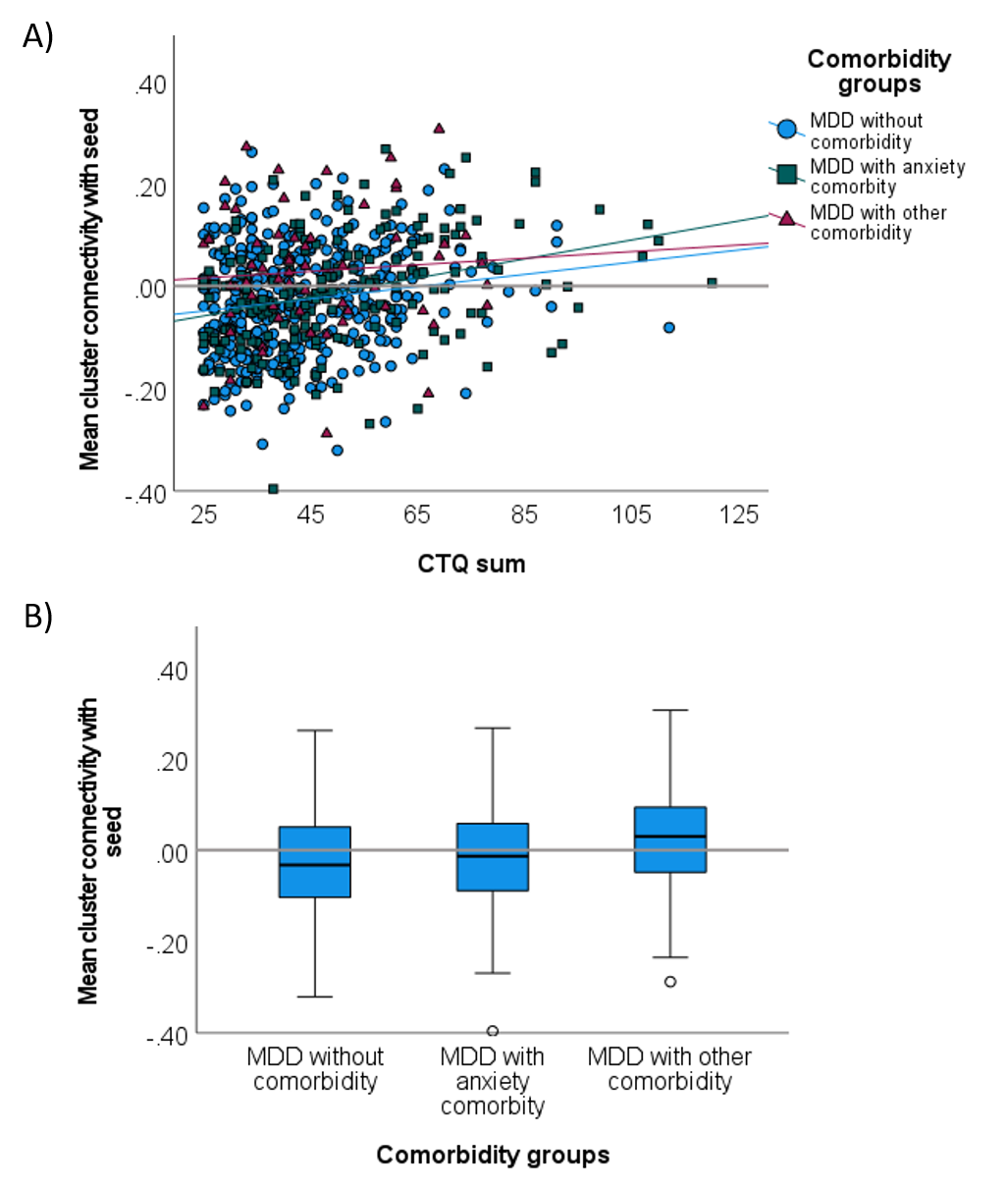


Figure S3. Extracted cluster connectivities between the right amygdala seed ROI and the dorsolateral prefrontal cluster across comorbidity groups within the major depressive disorder sample. A) Association between CTQ sum and cluster connectivity across comorbidity groups. B) Boxplot of cluster connectivity across comorbidity groups. The gray horizontal line represents a connectivity level of zero. MDD, major depressive disorder; CTQ, childhood trauma questionnaire.
